# Development of an artificial intelligence prediction model for moderate-to-severe COPD exacerbations using continuous multiple unobtrusive sensors: protocol of a multicentre prospective observational study

**DOI:** 10.1136/bmjresp-2025-003942

**Published:** 2026-06-04

**Authors:** Roger Vásquez-Andrade, Victoria Alcaraz-Serrano, Joren Buekers, Pasquale Bufano, Marco Laurino, Alessandro Celi, Henrik Watz, Joaquim Gea, Nicola Carbonaro, Alessandro Tognetti, Judith Garcia-Aymerich

**Affiliations:** 1 Barcelona Institute for Global Health, Barcelona, Spain; 2 Universitat Pompeu Fabra, Barcelona, Spain; 3 Institute of Clinical Physiology, National Research Council, Pisa, Italy; 4 Department of Surgical, Medical, Molecular and Critical Area Pathology, University of Pisa, Pisa, Italy; 5 Velocity Clinical Research Germany, Ahrensburg, Germany; 6 Airway Research Center North (ARCN), German Center for Lung Research (DZL), University of Lübeck, Lübeck, Germany; 7 Servei de Pneumologia, Hospital del Mar, Hospital del Mar Research Institute, Barcelona, Spain; 8 MELIS Department, Universitat Pompeu Fabra, Barcelona, Spain; 9 Department of Information Engineering, University of Pisa, Pisa, Italy

**Keywords:** COPD Exacerbations, Telemedicine, COPD, Exercise

## Abstract

**Introduction:**

Exacerbations, impaired health-related quality of life (HRQoL) and reduced exercise capacity increase the risk of hospitalisations and death in chronic obstructive pulmonary disease (COPD). However, their monitoring relies on in-person assessments, potentially delaying early care. While smart sensing technologies can enable remote monitoring, their use in predicting disease worsening remains limited. The TOLIFE Clinical Study A (CSA) aims to develop an artificial intelligence (AI) model integrating clinical data with smart sensing devices data to predict exacerbation onset and changes in HRQoL, dyspnoea and exercise capacity in people with COPD.

**Methods and analyses:**

TOLIFE CSA is a longitudinal observational study that will recruit 150 clinically stable people with COPD from three clinical sites in Spain, Italy and Germany. Over 1 year, participants will attend quarterly in-person visits to collect clinical data, while being continuously monitored using six unobtrusive smart sensing devices collecting daily metrics calculated from triaxial acceleration, angular velocity, photoplethysmogram, sound intensity, changes in latitude and longitude, ambient light intensity, biomechanical pressure and respiratory airflow parameters. Clinical outcomes are exacerbation onset through medical records; 3-month changes in HRQoL through the COPD Assessment Test and the Clinical COPD Questionnaire; 3-month changes in dyspnoea severity through the modified Medical Research Council Dyspnoea Scale; and 6-month changes in functional exercise capacity through the 6-minute walk test. We will train, internally validate and test AI-based models (Random Forests, XGBoost, multilayer perceptrons, cumulative link model and standard classification model) to predict clinical outcomes.

**Ethics and dissemination:**

Ethical approval was issued for all sites by the Ethical Commission (EC) of the Medical Association of Schleswig-Holstein (Bad Segeberg; vote 074/23 ff), EC of the Tuscany Region–North West Area (Pisa; vote CET10/2023) and EC of Parc de Salut Mar (Barcelona; vote 2023/11230). All participants will sign a written informed consent.

**Trial registration number:**

NCT06172712.

**WHAT IS ALREADY KNOWN ON THIS TOPIC**

- One barrier to early intervention in chronic obstructive pulmonary disease (COPD) is the reliance on interspersed in-person assessments to monitor key health outcomes. While smart sensing technologies are increasingly used for remote monitoring, their potential to support early intervention remains largely unexplored.

**WHAT THIS STUDY ADDS**

- This study aims to train, internally validate and test models to predict the onset of exacerbations and changes in health-related quality of life (HRQoL), dyspnoea severity and exercise capacity, with the goal of early intervention and attenuation of COPD progression.

**HOW THIS STUDY MIGHT AFFECT RESEARCH, PRACTICE OR POLICY**

- The findings will provide the development of a prediction tool that guides physicians in providing early, targeted care based on individual prediction estimates. It also fills a gap in the scientific evidence on prediction models for exacerbation onset and changes in HRQoL, dyspnoea severity and exercise capacity.

## Introduction

 Reducing symptoms, improving exercise capacity and preventing exacerbations are key treatment goals in chronic obstructive pulmonary disease (COPD).^1^ Exacerbations are particularly significant as they worsen health-related quality of life (HRQoL), reduce exercise capacity and increase the risk of future exacerbations, hospitalisations and death.^2^,^5^ Current guidelines recommend at least one annual visit for moderate-to-severe COPD and two for very severe cases to monitor disease progression, ideally including assessment of symptoms and exercise capacity.^6^ However, relevant changes in dyspnoea, HRQoL, exercise capacity or other clinical outcomes may occur between visits and remain undetected. Unfortunately, no tools are currently available for continuous monitoring of these key COPD health outcomes that would allow early identification of disease worsening and subsequent care.

Remote patient monitoring is a promising strategy for proactive COPD care, where sensor-based technologies are central to continuous data collection,^7^ and artificial intelligence (AI) is key to its subsequent integration and analysis. Sensor-based technologies, either worn on the body or placed in the home environment, continuously collect a wide range of physiological and behavioural data to support remote health assessment and potentially enable early detection of clinical deterioration.^8^ The advantages are amplified given that in respiratory disease, passive monitoring strategies (eg, wearable oxygen saturation device) achieve superior patient adherence compared with active methods (eg, manual pulse oximeter recording).^9^ Moreover, AI methods have shown potential to outperform traditional approaches in predicting COPD-related outcomes, particularly when integrating multiple data sources. For example, a random forest classifier outperformed a rule-based symptom detection method in predicting exacerbations within 3 days (area under the receiver operating characteristic curve (AUROC) 0.727 compared with 0.655),^10^ and gradient boosting models have outperformed exacerbation history alone in predicting severe exacerbations (AUROC 0.82 compared with 0.68).^11^ Moreover, combining clinical, environmental and lifestyle data improved 7-day exacerbation prediction (AUROC 0.935 compared with 0.835 when clinical data were excluded).^12^ However, there is still limited evidence on whether sensor-derived data can effectively predict key COPD outcomes or whether constructs increasingly associated with COPD progression, such as gait patterns^13^ and sleep quality,^14^ can effectively improve these predictions.

The TOLIFE Clinical Study A (CSA) aims to train, internally validate and test a modular AI-based analytical framework that integrates clinical data from standardised tests with continuously gathered daily-life data from six unobtrusive devices (smartphone, smartwatch, portable spirometer, smartshoes, smart mattress cover and environmental box). The primary prediction target is the timing of onset of moderate-to-severe exacerbations. Separate patient-specific models will address changes in HRQoL, dyspnoea severity and functional exercise capacity.

## Methods and analysis

We follow the Strengthening the Reporting of Observational Studies in Epidemiology (STROBE) guidelines within this manuscript.^15^ The study protocol represents version 2, June 2024.

### Study design

**Figure 1:**
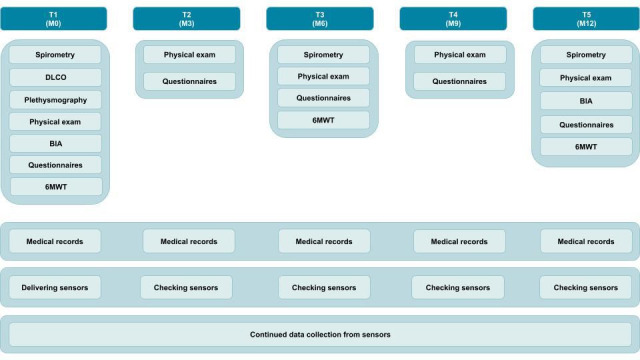
Overview of study design and data collection schedule in the TOLIFE CSA study. 6MWT, 6-minute walk test; BIA, bioelectrical impedance analysis; CSA, Clinical Study A; DLCO, diffusing capacity for carbon monoxide; M0–M12, month 0–month 12; T1–T5, visit 1–visit 5.

TOLIFE CSA is a longitudinal observational study that will run from May 2024 to April 2026. It includes a baseline visit (visit T1), followed by four follow-up visits every 3 months (visits T2, T3, T4 and T5, respectively) and continuous monitoring using smart sensing devices over 12 months (figure 1). All visits will take place in person at each participating clinical site.

### Participants

We will recruit people with stable COPD from three tertiary hospitals: Azienda Ospedaliero Universitaria Pisana (Pisa, Italy), Hospital del Mar (Barcelona, Spain) and the Pulmonary Research Institute (Großhansdorf, Germany). COPD will be defined according to the American Thoracic Society (ATS) and European Respiratory Society (ERS) guidelines as a postbronchodilator forced expiratory volume one second (FEV_₁_) to forced vital capacity (FVC) ratio of less than 0.70.^16^ Inclusion criteria are: (1) individuals with moderate-to-severe COPD (FEV_₁_ <80% of predicted) and a documented history of at least one moderate or one severe exacerbation (see definitions below) in the 12 months before enrolment; (2) age 40 years or older; (3) ability to walk at least 4 m independently, with or without walking aids; (4) availability for repeated study visits over 12 months; (5) willingness to use smart sensing devices; and (6) ability to read and write in the primary language(s) of the study site. Exclusion criteria are: (1) a history of myocardial infarction, hospitalisation for unstable angina, stroke, coronary artery bypass graft, percutaneous coronary intervention or implantation of a cardiac resynchronisation therapy device within 3 months before providing informed consent; (2) uncontrolled congestive heart disease (New York Heart Association class >3); (3) primary respiratory diseases other than COPD; (4) a history of major lung surgery (eg, lung transplant); (5) a major respiratory infection or exacerbation within 4 weeks before study inclusion or lung volume reduction within 6 months before study inclusion; (6) active treatment for cancer or other malignant diseases that could impact quality of life and adherence to the study; (7) a mental disorder that impedes effective participation; (8) a severe disease limiting survival to 1 year; (9) severe cognitive impairment (Mini-Mental State Examination <18); (10) significant mobility limitations unrelated to COPD; and (11) inability to follow study procedures due to language barriers or psychological disorders.

The target sample size was determined based on the feasibility of recruiting eligible participants within the funded study timeframe. We aim to recruit 150 participants, each contributing an average of 360 daily measurements, for an expected total of approximately 54 000 daily observations. For the primary outcome, onset of moderate-to-severe exacerbations, we anticipate at least 106 events, based on data from a large cohort study showing that individuals with a history of one moderate exacerbation experience an average of 0.71 subsequent exacerbations after a year of follow-up.^17^ According to the events-per-variable rule of thumb, predictive models should ideally include no more than one candidate predictor per 10 outcome events to reduce the risk of overfitting. With our sample size, there is a risk of prediction uncertainty and instability because we might have too many predictor variables relative to the anticipated number of outcome events. To mitigate this risk, we will perform a variable selection process to identify and retain only the most significant risk factors before developing the final predictive models. However, in our context, where the purpose is to provide early standard care, even models with moderate instability can still be useful for guiding timely decisions.^18^

### Measurements

We will collect clinical data at scheduled timepoints throughout the study, with assessment frequency reflecting each measure’s expected rate of change and the need to balance data collection with participant burden. The daily-life data from the TOLIFE Kit will be collected continuously during the study (figure 1).

#### Clinical outcomes

We will collect data on moderate (requiring treatment with short-acting bronchodilators and oral corticosteroids, with or without antibiotics) and severe (requiring hospitalisation or an emergency room visit) exacerbations from medical records at each study visit, using the Global Initiative for Chronic Obstructive Lung Disease (GOLD) guidelines to define severity.^1^ For each exacerbation, we will additionally record the start and end dates of treatment, emergency room visits, hospital, home or intensive care unit admissions, the need for mechanical ventilation and any exacerbation-related deaths.

We will assess HRQoL using the validated German, Italian and Spanish translations of the COPD Assessment Test (CAT)^19^ and the Clinical COPD Questionnaire (CCQ)^20^ at every study visit. The CAT score ranges from 0 to 40, with higher scores (>10) indicating a more severe impact of COPD on a patient’s life. The CCQ is scored in three subdomains (symptoms, functional state and mental state) and a total score, all of them ranging from 0 to 6, with higher scores indicating worse health status.

We will assess dyspnoea severity using the modified Medical Research Council (mMRC) Dyspnoea Scale at every visit,^21^ which measures the level of breathlessness on a scale from 0 (breathlessness with strenuous exercise) to 4 (breathlessness with minimal effort).

We will assess functional exercise capacity using the 6-minute walk test (6MWT) according to ERS/ATS guidelines^22^ at visits T1, T3 and T5 (figure 1). The 6MWT is a self-paced walking test in which participants walk as far as possible in 6 min along a flat corridor, with a length of 30 m at Azienda Ospedaliero Universitaria Pisana and Hospital del Mar, and 20 m at the Pulmonary Research Institute. Before and after the test, we will measure oxygen saturation, heart rate, blood pressure and the Borg dyspnoea and fatigue scores. During the test, we will record the number of breaks and their duration. After the test, we will monitor recovery by measuring oxygen saturation and heart rate every minute for 5 min, and calculate the 6-minute walk distance (6MWD).

#### Predictors

##### Sensor data

The TOLIFE Kit includes six unobtrusive commercial and research prototypes that will be used continuously during the study (table 1). At each visit, participants are instructed to use all devices according to recommended minimum usage protocols, with the smartphone being mandatory (table 2). However, participants retain the discretion to refrain from using certain devices. The participants can also prospectively indicate periods between visits when they will not be using (some of) the selected devices.

**Table 1:** Embedded sensors and devices and planned raw data to be collected

Sensors	Device	Raw data
Accelerometer	Samsung Galaxy Watch 5, Samsung M13, smartshoes, smart mattress cover	Triaxial acceleration (acceleration in x, y and z axes)
Gyroscope	Samsung Galaxy Watch 5, Samsung M13, smartshoes	Angular velocity (rotation in x, y and z axes)
Photoplethysmography (PPG) sensor	Samsung Galaxy Watch 5, Smart One Oxi	Photoplethysmogram
Microphone	Samsung Galaxy Watch 5, Samsung M13, environmental box	Sound intensity
GPS*	Samsung Galaxy Watch 5, Samsung M13	Latitude and longitude
Light sensor	Samsung Galaxy Watch 5, environmental box	Light intensity
Pressure sensor at 3 points	Smartshoes	Biomechanical pressure
Pressure sensor at 40 points	Smart mattress cover	Biomechanical pressure
Air quality sensor	Environmental box	Air quality, temperature and humidity
Turbine flow sensor	Smart One Oxi	Respiratory airflow

* Although these devices include a GPS sensor, the absolute position of the participant will not be tracked. We will use a derivative parameter that will not allow us to identify the absolute position of the participants.

**Table 2:** Recommended usage time for the TOLIFE Kit

Device	Recommended use
Samsung Galaxy Watch 5	It should be worn at all times and charged overnight.
Samsung M13	It should be kept in a pocket when outside and may rest on any surface at home. It should be charged overnight.
Smartshoes	It should be worn for 60 min/day and charged overnight.
Smart mattress cover	It should remain always activated and used every night for sleep.
Environmental box	It should remain always activated.
Smart One Oxi	Should be used once a week.

Three of them are commercial devices, including the smartwatch Samsung Galaxy Watch 5, the Samsung M13 smartphone and the Smart One OXI spirometer. The smartwatch and smartphone, unlike many other commercial devices, allow direct control of their sensors and provide access to raw data via software development kits, enabling the creation of applications for data collection and sensor management. These devices will gather raw data using their built-in sensors: both the smartwatch and smartphone are equipped with accelerometers, gyroscopes, microphones and GPS. Additionally, the smartwatch contains a photoplethysmography (PPG) sensor and an ambient light sensor. The Smart One OXI spirometer will collect data on pulmonary airflow and uses a PPG sensor. The specific raw data collected by each sensor are detailed in table 1.

The research prototype devices include smart shoes, a smart mattress cover and an environmental unit. The smart shoes are equipped with embedded sensors in the insoles, including accelerometers, gyroscopes and pressure sensors. The smart mattress cover is designed to be placed on top of the participant’s mattress at the thoracic level and collects raw data using accelerometers and pressure sensors.^23^ The environmental unit will be placed in the participant’s bedroom and is equipped with a microphone, ambient light sensor and air quality sensor. As with the commercial devices, the specific raw data collected by these sensors are listed in table 1.

##### Clinical data

We will perform standardised procedures to obtain (1) weight and height from a physical examination at visit T1, from which we will calculate body mass index; (2) total body water, fat mass and fat-free mass from bioelectrical impedance analysis at visits T1 and T5; (3) postbronchodilator FEV_1_ and FVC from forced spirometry^16^ at visits T1, T3 and T5; (4) tidal volume, residual volume, expiratory reserve volume, inspiratory reserve volume, inspiratory capacity, functional residual capacity, vital capacity and total lung capacity from body plethysmography^24^ at visit T1; and (5) diffusing lung capacity for carbon monoxide and alveolar volume from the single-breath carbon monoxide uptake test^25^ at visit T1.

Participants will respond to interviewer-administered validated questionnaires at every visit that will provide data on (1) individual sociodemographic characteristics (only at T1), including employment, marital status, living arrangement, education level, ethnicity; (2) smoking history (status, intensity and duration); (3) sleep quality from the Pittsburgh Sleep Quality Index^26^; (4) anxiety and depression symptoms from the Hospital Anxiety and Depression Scale^27^; and (5) general HRQoL from the 5-level EuroQol 5-Dimension Questionnaire.^28^

Lastly, we will collect data on comorbidities and pharmacological and non-pharmacological treatments from medical records at every visit.

### Statistical analysis

The characteristics of the participants will be summarised using means and SD for continuous variables and frequencies and percentages for categorical variables.

The collected data will be processed through a modular modelling architecture. Low-level models will convert raw sensor outputs from each device (continuous physiological signals, discrete events and questionnaire-derived outcomes) into standardised physiological and behavioural indicators. These indicators, integrated with clinical variables, will serve as inputs to the higher level predictive models.

To predict the onset of moderate-to-severe COPD exacerbations, both rule-based and machine learning approaches will be compared during development, with the single best performing strategy in terms of predictive accuracy and clinical interpretability selected for the final system. The model will output a continuous daily probability score estimating exacerbation risk within a given future time window, rather than a binary prediction. To predict changes in 6MWD, CAT, CCQ and mMRC outcomes, we will model their levels and their changes over time, both as continuous and dichotomised according to minimal importance difference (following the most recent literature cut-offs at the time of data analysis), using a cumulative link model or a standard classification model, respectively. Predictors will consist of sensor-derived indicators, aggregated over different temporal windows (eg, 7, 14, 21 and 30 days for the exacerbation models; 21-day summaries for the 6MWD, CAT, CCQ and mMRC models), together with clinical variables.

For all models, statistical inference will rely on a clustered bootstrap approach with resampling at the participant level to account for repeated measurements; a group-based validation strategy will be adopted, with training and testing sets separated at the clinical centre level to prevent information leakage and assess cross-site generalisability; and performance will be evaluated at both the cohort and patient-specific levels, assessing whether the models reliably detect meaningful deviations relative to each participant’s baseline over time. Standard classification metrics will be reported for all models using the Ranked Probability Score when applicable. For the 6MWD, CAT, CCQ and mMRC models, a leave-one-subject-out validation strategy will additionally be used with principal component analysis to address collinearity among predictors.

### Data quality control

Data collection will be conducted by trained assessors, all of whom are respiratory healthcare providers (nurses or physicians). To ensure standardised data collection, the technical coordinators (TC) and clinical study coordinators (CSC) will organise a training workshop that covers: (1) recruitment strategies like participant identification, eligibility criteria, study information delivery and informed consent administration, (2) instructions on administering questionnaires and clinical tests, with guidance on optimal timing and locations, (3) setting up and using the TOLIFE Kit devices, understanding their functionality and communicating key messages to participants and (4) use of the TOLIFE data collection platform, including logging in, uploading data and monitoring uploaded information. To strengthen these skills, a pilot study will be conducted in each clinical site to test the platform and evaluate the assessors’ performance in administering clinical tests and questionnaires.

During CSA data collection, the TC and CSC will be responsible for continuous data quality checks. The TC will ensure that devices are functioning correctly, data transmission is accurate and device usage aligns with the agreements made with participants during every visit. Meanwhile, the CSC will review downloaded data for inconsistencies and perform weekly cross-validation checks. The platform will automatically validate data by accepting only values within predefined ranges. Any issues identified through central monitoring will be compiled into a report and shared with each site monthly. Additionally, the clinical and technical teams will hold regular meetings to address any necessary improvements in the data collection process.

To ensure that device or usability issues are identified and resolved promptly, participants can contact the research team during working hours through a dedicated hotline, with contact details of their local principal investigator and research team provided in the Participant Information Sheet at enrolment. The TOLIFE data collection platform also generates automated alerts when no incoming data or low device usage is detected. On receiving an alert, the TC contacts the participant to troubleshoot, and issues that cannot be resolved locally are escalated to the technology partner for technical investigation and, if necessary, device replacement.

### Data management plan

The data collection process combines automated uploads of continuous smart sensing device data and manual uploads of clinical data by assessors during the visits, all directed to the TOLIFE data collection platform. The smartphone connects to a local router and receives data via Bluetooth from the smart shoes and spirometer. On the other hand, the smartwatch collects raw sensor data locally using an ad hoc application. Both the smartphone and smartwatch offload their data when they are plugged in for battery recharge. The smart mattress cover and environmental unit operate independently, collecting data through their sensors, storing it locally and uploading it whenever a connection is available. Clinical data, on the other hand, are gathered by authorised assessors during visits and uploaded directly to the platform.

The TOLIFE project employs a comprehensive, multilayered approach to data security, ensuring the confidentiality and integrity of sensitive participant information. Data from the three recruitment sites will be stored securely across two main infrastructures: Microsoft OneDrive, which provides institutional cloud services with user role management, and the TOLIFE Database, equipped with multilayer firewalls, real-time encryption and a backup system replicated to a secondary data centre for disaster recovery and ransomware protection. To protect personal data, pseudonymisation techniques will be used to separate personal identifiers from research data, with directly identifying information securely stored at clinical sites and scheduled for destruction after the study concludes. Final datasets will be stored in the Zenodo repository, which applies its own rigorous security protocols. This approach adheres to international standards such as International Standardisation Organisation 27001 and General Data Protection Regulation, safeguarding data throughout the project life-cycle.

### Patient and public involvement

A Patient Advisory Group provided input to help shape the study design, objectives, population characteristics, questionnaires and tests and the acceptability of smart sensing devices used in TOLIFE CSA. The Patient Advisory Group will continue to offer guidance on key topics, including identifying relevant smart sensing technology data and assisting in result interpretation and dissemination.

### Study status

Assessors’ training began in January 2024, totalling approximately 24 hours. Following ethical approval at all participating sites, participant recruitment was conducted from May 2024 to October 2025. As of October 2025, ninety-six participants have been recruited into the study. However, recruitment at Azienda Ospedaliero Universitaria Pisana has been delayed due to pending safety regulatory requirements for research prototype devices.

### Ethics and dissemination

All potential participants must sign an informed consent form before taking part in the study. Participants do not receive financial compensation for their participation. All study-related assessments and visits are conducted at no cost to participants, and the TOLIFE Kit devices are provided free of charge for the duration of the study. The study was registered at ClinicalTrials.gov on 15 December 2023 and titled ‘COPD Exacerbation Modelling Using Unobtrusive Sensors—the TOLIFE Clinical Study A (CSA)’ (ClinicalTrials.gov identifier: NCT06172712). Study results will be shared through peer-reviewed publications, conference presentations, social media and community engagement. The dissemination plan ensures findings are accessible to participants, healthcare professionals and the public, maximising their impact on COPD management.

## Discussion

COPD management is increasingly moving towards precision medicine. Initially, the GOLD guidelines relied solely on an FEV_1_-focused approach to guide treatment.^29^ Over time, this shifted to a patient-centred model, prioritising patients’ symptoms and exacerbation history.^30^ More recently, the guidelines have evolved to support personalised follow-up and introduce treatment strategies based on Treatable Traits.^31^ Still, challenges remain. Exacerbation history is the strongest predictor of future exacerbations,^32^ but COPD heterogeneity poses particular challenges, as patients with similar clinical profiles and exacerbation histories face different disease progression.^33^ Smart sensing technologies appear as promising complementary tools. These devices provide continuous, real-time monitoring of physiological parameters that can capture the clinical variability of COPD, allowing for personalised prediction estimates of exacerbations and early detection of changes in HRQoL, dyspnoea severity and exercise capacity.

### Clinical relevance

The TOLIFE CSA has potential clinical implications. First, by continuously monitoring physiological parameters, the system could complement scheduled follow-up by identifying participants whose condition is deteriorating between visits. This approach may help reduce care delays and support earlier intervention.^6 34^ Second, the data captured by the system could provide clinicians with additional context for their decisions, by flagging clinically meaningful changes. For instance, changes in metrics such as CAT/CCQ scores or 6MWT distance could alert clinicians to review treatment in line with local guidelines. Finally, the system continuously captures physiological parameters used in the Rome classification of exacerbation severity,^35^ now adopted by the GOLD 2026 guidelines^36^ (respiratory rate, heart rate, oxygen saturation), which could support remote severity assessment and help distinguish sustained deterioration from transient fluctuations. The clinical utility of these capabilities will require prospective validation.

### Strengths and limitations

The TOLIFE CSA study offers significant advantages. The multicentre design captures the variability of environment, behaviours and cultural differences across Western European patients with COPD, making predictions applicable across these populations. TOLIFE Kit devices will passively and continuously collect a vast amount of data, addressing the gaps in predictor availability.^37^ Compared with similar prediction models,^1238^,^40^ TOLIFE includes a larger cohort, longer follow-up and an event-based definition of exacerbations verified by respiratory specialists through medical records. However, some limitations exist. First, the current guidelines’ definitions of moderate and severe exacerbations rely on treatment with corticosteroids or hospitalisation,^1^ which may be influenced by healthcare system differences and clinical decision-making. Second, the study does not include relevant COPD biomarkers like eosinophil count or C-reactive protein levels, which are essential for COPD management decision-making.^41 42^ Third, ensuring adherence will be challenging, as technology illiteracy and discomfort with devices are common among people with COPD,^43^ which may impact data completeness. To address this, participants will choose devices they are most comfortable with at each visit, and their interaction with the devices will be minimal after the initial set-up. Fourth, data heterogeneity may result from impaired software updates at single and multi-device levels.^44^ However, the study includes commercial devices that allow the collection of raw data and are configured to prevent automatic software updates, ensuring complete control over algorithms. Fifth, compliance with the device usage protocol and technical reliability of the sensing devices may affect data completeness. App malfunctions, data loss or periods of non-compliance could result in gaps in the sensor-derived data streams. To mitigate this, the algorithms will be designed to handle missing data from any cause by adaptively combining overlapping measurements when available and by using temporal aggregation to reduce the impact of intermittent gaps on predictions. Sixth, there is a risk of prediction instability due to our limited sample size. To manage the clinical consequences of this, the model will function as a high-sensitivity tool. This approach aligns with the clinical principle of maximising net benefit in a low clinical threshold context,^18^ which is achieved by prioritising the detection of patients at risk over the avoidance of false alarms.

## Data Availability

Data sharing not applicable as no datasets generated and/or analysed for this study.
